# Intravitreal Vascular Endothelial Growth Factor Inhibitor Use and Renal Function Decline in Patients with Diabetic Retinopathy

**DOI:** 10.3390/ijerph192114298

**Published:** 2022-11-01

**Authors:** Shih-Hsiang Ou, Chun-Hao Yin, Tung-Ling Chung, Hsin-Yu Chen, Chien-Liang Chen, Jin-Shuen Chen, Po-Tsang Lee

**Affiliations:** 1 Division of Nephrology, Department of Internal Medicine, Pingtung Veterans General Hospital, Pingtung 900, Taiwan; 2 Division of Nephrology, Department of Internal Medicine, Kaohsiung Veterans General Hospital, Kaohsiung 813, Taiwan; 3 Faculty of Medicine, School of Medicine, National Yang Ming Chiao Tung University, Taipei 112, Taiwan; 4 Graduate Institute of Clinical Medicine, Kaohsiung Medical University, Kaohsiung 807, Taiwan; 5 Department of Medical Education and Research, Kaohsiung Veterans General Hospital, Kaohsiung 813, Taiwan; 6 Institute of Health Care Management, National Sun Yat-sen University, Kaohsiung 804, Taiwan; 7 Faculty of Medicine, School of Medicine, National Defense Medicine Center, Taipei 114, Taiwan

**Keywords:** diabetic retinopathy, renal function decline, vascular endothelial growth factor inhibitors

## Abstract

Adverse renal effects of systemic vascular endothelial growth factor (VEGF) inhibitor treatment are well documented. We aimed to identify associations between intravitreal VEGF inhibitor use and renal function decline in patients with diabetic retinopathy. We included 625 patients with diabetic retinopathy for regular renal function follow-ups and grouped them according to intravitreal therapy (67 with and 558 without treatment). We used a generalized estimating equation model to identify renal function decline risk factors. Increased age (*p* = 0.02), insulin use (*p* = 0.01), hypertension (*p* < 0.01), and ischemic heart disease (*p* < 0.01) were associated with significantly decreased estimated glomerular filtration rates (eGFRs) in patients with diabetic retinopathy after 1-year follow-up. Compared to the control group, patients who received intravitreal VEGF inhibitor injections showed a declining eGFR trend in the repeated measurement model without statistical significance (*p* = 0.06). In subgroup analysis, patients with initial eGFR ≤ 30 mL/min/1.73 m^2^ who received intravitreal VEGF inhibitors had significantly decreased renal function (*p* < 0.01) compared to those without treatment. Intravitreal VEGF inhibitor injection was associated with renal function deterioration among patients with diabetic retinopathy and advanced chronic kidney disease. Strategies to monitor renal function after treatment should be considered in these high-risk populations.

## 1. Introduction

Vascular endothelial growth factor (VEGF) inhibitors can suppress neo-angiogenesis in malignancy and are widely used as an anti-neoplastic treatment for many solid tumors, including non-small cell lung cancer, breast cancer, and colorectal cancer [1,2]. However, intravenous administration of VEGF inhibitors can interrupt VEGF and related signaling homeostasis in other organs and lead to adverse effects. In terms of nephrotoxicity, side effects such as hypertension, proteinuria, glomerulonephritis, and vascular clotting events have been reported [3,4,5,6]. Therefore, the renal function of patients with kidney disease should be closely monitored after systemic VEGF inhibitor treatment.

The prevalence of diabetes mellitus is rising worldwide [7], leading to a rise in associated microvascular complications, such as nephropathy and retinopathy. Currently, diabetic retinopathy is the leading cause of vision loss in adults aged 20–74 years [8]. One of the major drivers in the pathophysiology of diabetic retinopathy is VEGF. Overexpression of VEGF induces retinal neovascularization and blood–retinal barrier disruption, resulting in proliferative diabetic retinopathy and diabetic macular edema, respectively [9,10]. Since VEGF plays an important role in the development of diabetic retinopathy, intravitreal VEGF blockade has become one of the major treatment options for this condition [11,12].

The therapeutic ophthalmic dose of VEGF inhibitors by intravitreal injection is approximately 150–400 times lower than the systemically used dose in oncology treatment [13,14]. However, previous studies have shown that intravitreally injected VEGF inhibitors can cross the blood–retinal barrier, enter the systemic circulation, and even affect the circulating VEGF levels in serum [15,16]. Whether intravitreal medication usage could lead to systemic side effects is a matter of concern. Some ophthalmic studies suggest that the risk of thromboembolic events is similar in patients with and without intravitreal VEGF inhibitor injection [17,18]. Contrarily, several reports indicated that the rates of cardiovascular and cerebrovascular events might be higher in participants who received treatment [19,20]. Results from previous studies on the influence of VEGF inhibitors on kidney function were also inconsistent. There have been some case reports of worsening proteinuria, development of glomerulonephritis, acute kidney injury, and even renal failure after intravitreal VEGF inhibitor injection [21]. However, a recent retrospective study in Japan did not report an increased risk of acute kidney injury after a short-term follow-up of 7–30 days following intravitreal VEGF injection in patients with diabetes and chronic kidney disease [22].

Previous studies documenting the association between intravitreal administration of VEGF inhibitors and renal complications are controversial. Studies on their long-term impact on renal function are also scarce. Considering that most patients required several courses of injections in a year, we compared the exact changes in renal function one year before and after starting treatment. Our present study aimed to investigate whether intravitreal administration of VEGF inhibitors was associated with the deterioration of renal function in patients with diabetic retinopathy and attempted to identify the precipitating factors.

## 2. Patients and Methods

### 2.1. Study Participants and Design

We conducted a retrospective cohort study that included patients with diabetic retinopathy at Kaohsiung Veterans General Hospital, Taiwan, between 1 January 2010 and 31 December 2019. Data on the participants were collected through chart review to investigate the risk of renal function deterioration. The ethics committee of Kaohsiung Veterans General Hospital approved the protocol (IRB No: KSVGH20-CT7-16) and supervised the study in accordance with the tenets of the Declaration of Helsinki (1975) and its later amendments (2013). The requirement of informed consent was waived due to the retrospective nature of this study.

Patients from the outpatient department who fulfilled the following criteria were included in the study: (i) diagnosed with diabetic retinopathy by the disease codes at the attending ophthalmologist clinic; (ii) aged ≥ 20 years; and (iii) received regular renal function follow-up (at least twice yearly in two consecutive years) after the confirmation of diabetic retinopathy diagnosis. The disease and diagnostic codes recorded in the registry of clinical visits and hospital care were based on the International Classification of Diseases, Ninth and Tenth Revisions, Clinical Modifications (ICD-9-CM and ICD-10-CM). Patients with diabetic retinopathy were defined as patients reporting at least three or more specific outpatient disease codes (ICD-9: 362; ICD-10: E11.3). The exclusion criteria were: (i) a diagnosis of end-stage renal disease or received renal transplantation and (ii) other disease-related renal abnormalities and confirmed glomerulopathy, interstitial nephritis, or tubulonephritis other than diabetic nephropathy.

### 2.2. Definition of Intravitreal VEGF Inhibitors Therapy

We categorized the enrolled patients into the following two groups: those who received intravitreal VEGF inhibitor treatment and those who did not. We defined the treatment group as those who received intravitreal injection therapy of VEGF inhibitors at least three times during a six-month course at the Department of Ophthalmology, Kaohsiung Veterans General Hospital. The agents included bevacizumab, aflibercept, or ranibizumab (Supplementary Table S1). Each patient was injected under sterile conditions with either 1.25 mg of bevacizumab, 0.5 mg of ranibizumab, or 2.0 mg of aflibercept into the vitreous cavity each time. The first time the intravitreal VEGF inhibitors were administered was the index date. The control group included patients with diabetic retinopathy without exposure to any VEGF inhibitor therapy before and during the observation period.

### 2.3. Assessment of Renal Function

The data of serial serum creatinine measurements were collected from the electronic medical records system. The estimated glomerular filtration rate (eGFR) was calculated using a modified equation from the Modification of Diet in Renal Disease study. The potential errors associated with single measurements were eliminated by enrolling patients with at least two separate renal function values in a defined period to obtain an average value. The study’s primary outcome was to compare the change in renal function between the baseline and follow-up measurements. In the treatment group, we compared the renal function change between one year before and after the index date. For comparison, the renal function among the control group was also tested more than twice in a year for two consecutive years, which was defined as the baseline value in the first year and the followed-up value in the second year. The follow-up duration of all patients was standardized to one-year after injection therapy or matched index date.

### 2.4. Evaluation of Other Covariates

Data on baseline demographics and underlying comorbidities were collected for each patient. Individuals with comorbidities, such as hypertension, hyperlipidemia, and ischemic heart disease, were confirmed before enrollment by their history as diagnosed by ICD codes in at least one admission code or three or more outpatient codes. Medication use, including non-steroidal anti-inflammatory drugs, angiotensin-converting enzyme inhibitors, angiotensin II receptor blockers, insulin, and oral hypoglycemic agents, was defined as receiving a prescription for more than 28 days during the study period. Contrast medium exposure history was established when at least one medical record about computed tomography with contrast medium, angiography, or radiology intervention using contrast medium was undertaken during the study period. All patients in the final analytical dataset had the most complete covariate data possible.

### 2.5. Statistical Analysis

Baseline characteristics were summarized for the entire analytical population and subgroups based on the intravitreal VEGF inhibitor usage. These were expressed as means (± standard deviations) or numbers (percentages). Differences between the groups were compared using independent Student’s *t*-tests and χ^2^-tests for normally distributed continuous and categorical variables, respectively. Non-parametric data were compared using the Mann–Whitney U test. In addition, significant determinants of renal function change were evaluated using univariate and multivariate linear regression models. The variables involving repeated measurements, including serum creatinine and eGFR measured before and after the index date, were analyzed using the generalized estimating equation (GEE) method. In GEE analysis, missing data were managed by the default setting of the statistical software and did not bias the calculation by its random nature [23,24]. Statistical significance was set at a two-tailed *p* < 0.05, and all statistical analyses were performed using the Statistical Package for Social Sciences, statistical software 22.0.0 (IBM Corp., Armonk, NY, USA).

## 3. Results

**Figure 1:**
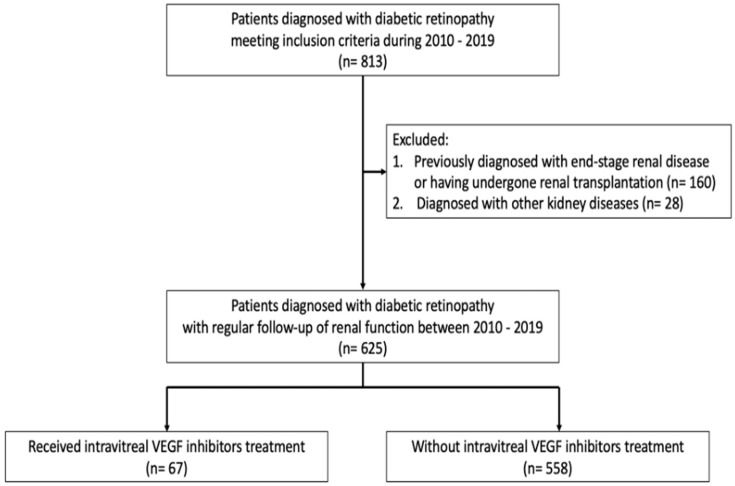
Flowchart describing enrollment of participants in the cohort study. VEGF, vascular endothelial growth factor.

During the study period, a total of 813 patients who were diagnosed with diabetic retinopathy fulfilled the inclusion criteria and were enrolled (Figure 1). After excluding patients with end-stage renal disease or renal transplantation (n = 160) and patients with a specific renal disease other than diabetic nephropathy (n = 28), 625 patients (383 male, 242 female) were included in the analysis. The mean age of the patients in the study was 62.1 ± 12.4 years old, and their average HbA1C was 7.9 ± 2.0%. Among them, 475 patients had a comorbidity of hypertension, 264 patients had dyslipidemia, and 123 patients had ischemic heart disease. The patients were then categorized into two groups according to the medical history of intravitreal VEGF inhibitor treatment.

### 3.1. Differences between Patients with Diabetic Retinopathy with or without Intravitreal VEGF Inhibitor Therapy

The clinical characteristics of the study groups are compared in Table 1. There were 67 and 558 patients with and without intravitreal agent injection, respectively. Compared to the control group, patients with intravitreal VEGF inhibitor treatment had lower mean glycated hemoglobin (HbA1c) levels (*p* = 0.02), higher prevalence of hyperlipidemia (*p* = 0.01), and higher prevalence of contrast medium exposure (*p* < 0.01). The difference in initial eGFR or follow-up eGFR was not significant between these two groups. The renal function change (follow-up eGFR minus initial eGFR) showed a sharp decline in the treatment group compared to that in the control group, although this change did not reach statistical significance (*p* = 0.07).

### 3.2. Renal Function Change in Patients with Diabetic Retinopathy

To evaluate the determinants of renal function change, we conducted a GEE analysis where all covariates from the preceding analysis were included as independent variables (Table 2). We collected data on factors associated with receiving intravitreal VEGF inhibitor treatment and the time effect as dependent variables to explore the interaction. Among the demographic factors examined, the following factors had significant associations: increased age was significantly associated with decreased eGFR (β = −0.229, confidence interval (CI): −0.42, 0.04; *p* = 0.02); elevated HbA1c was significantly associated with higher eGFR (β = 1.219, CI: 0.18, 2.26; *p* = 0.02); insulin users had larger eGFR decrements compared with non-insulin users (β = −4.788, CI: −8.19, −1.38; *p* = 0.01). The rapid renal function decline in insulin users is possibly caused by longer disease duration that may have resulted in insulin deficiency or poor baseline sugar control. When analyzed for comorbidities, patients with hypertension (β = −14.374, CI: −19.64, −9.1; *p* < 0.01) and ischemic heart disease (β = −13.457, CI: −18.38, −8.54; *p* < 0.01) had decreased eGFR. After adjusting for initial renal function and other covariates, patients who received intravitreal VEGF inhibitor treatment appeared to have a larger eGFR decrement when considering the time factor; however, this did not reach statistical significance (β = −3.045, CI: −6.15, 0.06; *p* = 0.06).

### 3.3. Stratification Analysis of Renal Function Change in Patients with Diabetic Retinopathy

**Figure 2:**
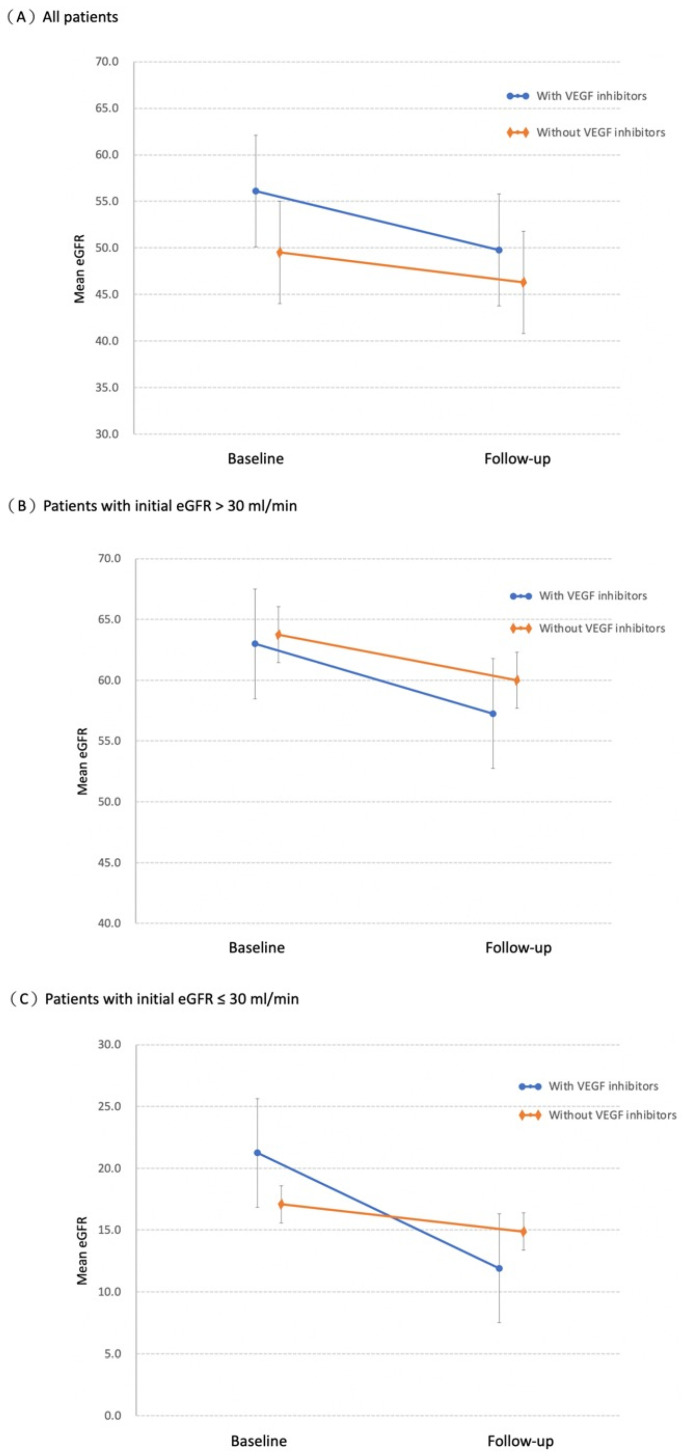
The change in renal function with time. (**A**) All patients: mean eGFR in the treatment group and control group at baseline and at 1-year follow-up. (**B**) Patients with initial eGFR > 30 mL/min/1.73 m^2^: mean eGFR in the treatment group and control group at baseline and at 1-year follow-up. (**C**) Patients with initial eGFR ≤ 30 mL/min/1.73 m^2^: mean eGFR in the treatment group and control group at baseline and at 1-year follow-up. Bars represent 95% confidence intervals. eGFR, estimated glomerular filtration rate; VEGF, vascular endothelial growth factor.

We then stratified patients with diabetic retinopathy into eGFR > 30 mL/min/1.73 m^2^ (n = 444) and eGFR ≤ 30 mL/min/1.73 m^2^ (n = 181) subgroups based on their initial renal function (Supplementary Table S2). The results of the repeatedly measured eGFR by respective grouping are shown in Figure 2. Each group was re-analyzed by the previous GEE method to examine the impact of treatment (Table 3). After adjusting for initial renal function, other covariates, and the time effect, intravitreal VEGF inhibitor administration did not affect the change in eGFR in patients with an initial eGFR > 30 mL/min/1.73 m^2^ (β = −1.984, CI: −5.67, 1.70; *p* = 0.29). In comparison, in patients with an initial eGFR ≤ 30 mL/min/1.73 m^2^, intravitreal VEGF inhibitor usage was significantly associated with renal function decline (β = −7.129, CI: −10.43, −3.82; *p* < 0.01).

## 4. Discussion

The current observational cohort study aimed to determine whether the use of intravitreal injection of VEGF inhibitors is associated with the deterioration of renal function in patients with diabetic retinopathy by repeated measurement analysis. Our data showed that patients who received intravitreal VEGF inhibitors tended to have a larger eGFR decrement after adjusting for other confounding factors than those without VEGF inhibitor use. After stratification by initial renal function, we found that the treatment was significantly associated with renal function decline in patients with initial eGFR ≤ 30 mL/min/1.73 m^2^. This result implies that advanced chronic kidney disease is an independent risk factor of kidney damage caused by intravitreal VEGF inhibitor therapy in patients with diabetic retinopathy.

VEGF plays a crucial role in maintaining normal renal function. In the kidneys, VEGF is mainly released from podocytes and activates VEGF receptor 2 on glomerular capillary endothelial cells, which maintains the integrity of endothelial fenestrations and the resultant glomerular barrier function [25,26]. When the intraglomerular VEGF signaling balance is dysregulated, regardless of upregulation or downregulation, it might be associated with endothelial dysfunction and podocyte dysregulation [27,28]. A previous animal study showed that loss of VEGF expression in podocytes results in proteinuria, hypertension, and renal thrombotic microangiopathy [4]. Similar renal side effects were also reported when VEGF inhibitors were used systemically in humans, including worsening of proteinuria, hypertension, glomerulonephritis, acute kidney injury, and even rarely thrombotic thrombocytopenic purpura [3,5,6,29]. Previous reports mostly suggested that intravitreal medication administration is a local treatment with barely any systemic side effects. However, Avery et al. reported that the systemic absorption of intravitreal VEGF inhibitors was greater and more substantial than previously reported [15,30]. Several studies also showed that systemic VEGF levels are significantly inhibited following the intravitreal injection of various VEGF inhibitors even after several weeks, although the clinical significance of this finding is still unclear [19,31,32,33]. Once the systemically absorbed drug concentration gradually accumulates and is eventually high enough to affect intraglomerular VEGF, it may cause nephrotoxicity. Therefore, long-term follow-up studies are required to investigate the real impact of this therapy on kidney function.

Although adverse renal effects caused by systemic treatment with VEGF inhibitors are well documented, the association between intravitreal VEGF inhibitor therapy and kidney injury is still debated. In 2015, the Diabetic Retinopathy Clinical Research Network reported a randomized clinical trial to compare the effectiveness between different intravitreal VEGF inhibitor therapies, which found that 11–12% of participants in each treatment group developed self-reported adverse renal effects during the 1-year follow-up [34]. A retrospective study in Japan compared the change of renal function in patients with diabetes and chronic kidney disease before and after intravitreal administration of VEGF inhibitors; the result suggested that the therapy is not associated with renal function deterioration during the 7–30-day period [22]. However, the follow-up duration was short, limiting the accumulation effects of VEGF inhibitors following their chronic use, which mostly lasts months. Similarly, another cohort study aimed to assess the effect of multiple intravitreal VEGF inhibitor injections on renal function in patients with diabetic retinopathy [35]. Although the finding showed that the injection numbers did not alter the rate of renal function decline over the 31-month period, the percentage of participants with an eGFR < 60 mL/min increased from 26% at baseline to 39% during the follow-up. The clinical significance of this finding could not be substantiated because of the lack of a comparative control group. Based on our findings, it is necessary to regularly monitor renal function in patients with diabetes with eGFR ≤ 30 mL/min/1.73 m^2^ and receiving intravitreal VEGF inhibitor injections.

There are several reasons for the vulnerability to renal damage in patients with advanced kidney disease using VEGF inhibitors. One possible reason is the higher incidence of comorbidities, including hypertension and coronary artery disease, in patients with advanced renal impairment. Patients with these chronic diseases usually have a higher risk of developing acute kidney injury [36,37,38,39]. Another reason is the consequence of reduced renal reserve following an insult leading to renal injury [40]. To minimize the fluctuation of serum creatinine levels during the treatment course, we used the average serum creatinine over a year to represent eGFR and reduced the bias. For differences in the timing of the blood test and the tracking interval of each participant, we used the repeated measurement analysis by the GEE model to compare the change in renal function. A control group with a diagnosis of diabetic retinopathy but not receiving intravitreal VEGF inhibitor injection was included as a comparison group because the renal function of patients with diabetic retinopathy may gradually decline over time. This helped to assess whether intravitreal VEGF inhibitor therapy results in additional kidney damage. We found that intravitreal VEGF inhibitor injection was significantly associated with renal function decline in patients with initial eGFR ≤ 30 mL/min/1.73 m^2^ during the one-year follow-up. Our study is the first report to support that intravitreal VEGF inhibitors should be used with caution in the high-risk group to avoid significant nephrotoxicity. One of the patient-related risk factors is advanced chronic kidney disease. Dose adjustment, cessation of therapy, or switching to a lower systemic potency agent could be considered as possible therapeutic options under these circumstances.

There are some limitations to the current study. First, the number of patients with intravitreal therapy and regular renal function check-ups in this retrospective study was relatively small. However, the power calculation was still more than 80% in overall or stratification analysis, implying that the sample size was sufficiently high to support the results. Second, we did not measure the times and frequency of intravitreal therapy, so we could not determine the dose effect for each participant. However, according to Taiwan’s health insurance application regulation, the treatment dose applied for diabetic retinopathy in the first year is around 3–5 doses. Third, we did not analyze the diabetic disease duration, medication compliance, and body mass index, which possibly influenced the renal function in patients with diabetes. We also did not explore other drugs that might affect renal function, such as diuretics or mineralocorticoid receptor antagonists. Fourth, we did not perform a renal biopsy to confirm the diagnosis of diabetic nephropathy, which is mostly diagnosed clinically in practice. Finally, we did not evaluate the association between the severity of retinopathy and the use of intravitreal VEGF inhibitors. Furthermore, we did not record and compare our results with alternative treatments for retinopathy. These limitations may warrant a more rigorous prospective study to explore these factors.

## 5. Conclusions

In conclusion, our study revealed that intravitreal VEGF inhibitor injection was independently and significantly associated with renal function decline in patients with diabetic retinopathy with advanced chronic kidney disease. Therefore, careful renal function monitoring and follow-up strategies or switching to another therapy should be considered in this high-risk group to avoid further nephrotoxicity.

## Figures and Tables

**Table 1:** Comparison of clinical characteristics among patients with diabetic retinopathy.

Parameter	Total	With VEGF Inhibitors	Without VEGF Inhibitors	*p*-Value
	N = 625	N = 67	N = 558	
Sex—male, N (%)	383 (61)	46 (69)	317 (57)	0.06
Age, mean ± SD	62.1 ± 12.4	60.3 ± 12.3	62.2 ± 12.3	0.24
HbA1c (%), mean ± SD	7.9 ± 2.0	7.5 ± 2.1	8.0 ± 2.0	0.02
Insulin user	305 (49)	37 (55)	268 (48)	0.35
Comorbidity				
Hypertension	475 (76)	51 (76)	424 (76)	0.98
Hyperlipidemia	264 (42)	38 (57)	226 (41)	0.01
Ischemic heart disease	123 (20)	10 (15)	113 (20)	0.30
Medication and exposure				
Contrast medium	134 (21)	28 (42)	106 (19)	<0.01
NSAID	475 (76)	52 (78)	423 (76)	0.74
ACEI/ARB	447 (72)	51 (76)	396 (71)	0.38
Renal function				
Initial creatinine (mg/dL)	2.1 ± 1.8	2.0 ± 2.0	2.1 ± 1.8	0.56
Initial eGFR (mL/min)	50.2 ± 28.3	56.1 ± 24.7	49.5 ± 28.6	0.07
Follow-up creatinine (mg/dL)	2.6 ± 2.5	2.6 ± 3.1	2.6 ± 2.4	0.97
Follow-up eGFR (mL/min)	46.6 ± 28.8	49.8 ± 26.8	46.2 ± 29.1	0.34
Renal function change (Follow-up eGFR–Initial eGFR)	−3.6 ± 12.9	−6.3 ± 12.3	−3.3 ± 12.9	0.07

Abbreviations: VEGF, vascular endothelial growth factor; SD, standard deviation; HbA1c, glycated hemoglobin; NSAID, non-steroidal anti-inflammatory drug; ACEI, angiotensin-converting enzyme inhibitors; ARB, angiotensin receptor blockers; eGFR, estimated glomerular filtration rate.

**Table 2:** Generalized estimating equation analysis for patients with diabetic retinopathy with repeatedly measured eGFR values.

Variables	Estimated Value	(95% CI)	*p*-Value
Inception (Initial eGFR)	75.606	(59.65; 91.56)	<0.01
Sex—male	2.460	(−1.55; 6.46)	0.23
Age	−0.229	(−0.42; −0.04)	0.02
HbA1c	1.219	(0.18; 2.26)	0.02
Insulin user	−4.788	(−8.19; −1.38)	0.01
Comorbidity			
Hypertension	−14.374	(−19.64; −9.10)	<0.01
Hyperlipidemia	−2.201	(−6.34; 1.93)	0.30
Ischemic heart disease	−13.457	(−18.38; −8.54)	<0.01
Medication and exposure			
Contrast medium	−0.466	(−5.29; 4.36)	0.85
NSAID	1.841	(−2.88; 6.56)	0.44
ACEI/ARB	−4.184	(−9.20; 0.83)	0.10
With anti-VEGF × Time effects	−3.045	(−6.15; 0.06)	0.06

Abbreviations: eGFR, estimated glomerular filtration rate; CI, confidence interval; HbA1c, glycated hemoglobin; NSAID, non-steroidal anti-inflammatory drug; ACEI, angiotensin-converting enzyme inhibitors; ARB, angiotensin receptor blockers; VEGF, vascular endothelial growth factor.

**Table 3:** Generalized estimating equation for patients with diabetic retinopathy with repeatedly measured eGFR values by stratification analysis.

	Total, n = 625 (100%)			Initial eGFR > 30 mL/min/1.73 m^2^, n = 444 (71%)			Initial eGFR ≤ 30 mL/min/1.73 m^2^, n = 181 (29%)		
Variables	Adjusted Estimated Value *	(95% CI)	*p*-Value	Adjusted Estimated Value *	(95% CI)	*p*-Value	Adjusted Estimated Value *	(95% CI)	*p*-Value
Anti-VEGF use									
Without	Reference			Reference			Reference		
With	7.054	(1.28; 12.82)	<0.01	−1.132	(−6.28; 4.02)	0.67	5.46	(1.29; 9.63)	0.01
Time effects									
Baseline	Reference			Reference			Reference		
Follow-up	−3.27	(−4.34; −2.20)	<0.01	−3.742	(−5.12; −2.36)	<0.01	−2.203	(−3.74; −0.66)	0.01
With anti-VEGF ×Time effects	−3.045	(−6.15; 0.06)	0.06	−1.984	(−5.67; 1.70)	0.29	−7.129	(−10.43; −3.82)	<0.01

* Adjusted for sex, age, HbA1c, insulin use, comorbidities, medication, and exposure listed in Table 1. Abbreviations: eGFR, estimated glomerular filtration rate; CI, confidence interval; VEGF, vascular endothelial growth factor; HbA1c, glycated hemoglobin.

## Data Availability

The data used to support the findings of this study are available from the corresponding author upon reasonable request.

## Supplementary Materials

The following supporting information can be downloaded at https://www.mdpi.com/article/10.3390/ijerph192114298/s1, Supplementary Table S1: The anatomical therapeutic chemical (ATC) classification codes for intravitreal vascular endothelial growth factor inhibitor agents; Supplementary Table S2: Demographic difference of stratification analysis by initial renal function between diabetic retinopathy patients.
