# Supplementary material for: Intravitreal Vascular Endothelial Growth Factor Inhibitor Use and Renal Function Decline in Patients with Diabetic Retinopathy
Source: Int J Environ Res Public Health. 2022 Nov 1;19(21):14298. doi: 10.3390/ijerph192114298 (PMC9657653; doi:10.3390/ijerph192114298)
Supplement: Supplementary file 1 [file ijerph-19-14298-s001.zip › ijerph-1978200-supplementary.pdf]

Supplementary Table

Table S1. The Anatomical Therapeutic Chemical (ATC) classification codes for intravitreal vascular endothelial growth factor inhibitor agents

Medication	ATC codes
Bevacizumab	L01XC07
Aflibercept	S01LA05
Ranibizumab	S01LA04

Table S2. Demographic difference of stratification analysis by initial renal function between diabetic retinopathy patients

Parameter	Total	Initial eGFR > 30 ml/min	Initial eGFR ≤ 30 ml/min	p-Value
	n = 625	n =444	n =181	
Sex - male	363 (58)	262 (59)	101 (56)	0.46
Age, (years)	62.1 ± 12.4	62.01 ± 12.7	61.9 ± 11.4	0.84
HbA1C (%)	7.9 ± 2.0	8.2 ± 2.0	7.5 ± 1.9	<0.01
Insulin user	305 (49)	204 (46)	101 (56)	0.04
Comorbidity				
Hypertension	475 (76)	312 (70)	163 (90)	<0.01
Hyperlipidemia	264 (42)	180 (41)	84 (46)	0.18
Ischemic heart disease	123 (20)	56 (19)	67 (37)	<0.01
Medication and exposure				
Contrast	134 (21)	95 (21)	39 (22)	0.97
NSAID	475 (76)	333 (75)	142 (79)	0.36
ACEI/ARB	447 (72)	307 (69)	140 (77)	0.04
Renal function				
Initial creatinine (mg/dL)	2.1 ± 1.8	1.2 ± 0.4	4.2 ± 2.1	<0.01
Initial eGFR (mL/min)	50.2 ± 28.3	63.7 ± 21.9	17.3 ± 7.1	<0.01
Followed creatinine (mg/dL)	2.6 ± 2.5	1.4 ± 0.8	5.4 ± 2.9	<0.01
Followed eGFR (mL/min)	46.6 ± 28.8	59.7 ± 23.1	14.7 ± 10.8	<0.01
Renal function change (Followed eGFR – Initial eGFR)	-3.6 ± 12.9	-3.9 ± 13.8	-2.6 ± 10.8	0.23

Abbreviation: VEGF, vascular endothelial growth factor; HbA1C, glycosylated hemoglobin, type A1C; NSAID, non-steroidal anti-inflammatory drug; ACEI, angiotensin-converting enzyme inhibitors; ARB, angiotensin receptor blockers; eGFR, estimated glomerular filtration rate
